# HPLC Fingerprints for the Characterization of Walnuts and the Detection of Fraudulent Incidents

**DOI:** 10.3390/foods10092145

**Published:** 2021-09-10

**Authors:** Natasa P. Kalogiouri, Victoria F. Samanidou

**Affiliations:** Laboratory of Analytical Chemistry, Department of Chemistry, Aristotle University of Thessaloniki, 54124 Thessaloniki, Greece; kalogiourin@chem.auth.gr

**Keywords:** walnuts, HPLC-DAD, authenticity, food fraud, chemometrics

## Abstract

A high-pressure liquid chromatographic method coupled to diode array detector (HPLC-DAD) was developed for the determination of phenolic compounds that could be used as markers in authentication studies of walnuts belonging to the Chandler variety, originating from Bulgaria, Greece, and France. An ultrasound-assisted extraction (UAE) protocol applied in the extraction of phenolic compounds was optimized. The method was validated and the relative standard deviations (RSD%) of the within-day, and between-day assays was lower than 6.3 and 11.1, respectively, showing adequate precision, and good accuracy ranging from 86.4 (sinapic acid) to 98.4% (caffeic acid) for within-day assay, and from 90.1 (gallocatechin gallate) to 100.6% (gallic acid) for between-day assay. Eighteen phenolic compounds were determined belonging to the classes of phenolic acids and flavonoids. The quantification results were further processed with chemometrics, and a robust partial least square–discriminant analysis (PLS-DA) model was developed for the classification of the samples according to their geographical origin, proposing markers that could be used for the control of walnuts authenticity and the detection of fraudulent incidents.

## 1. Introduction

Food authenticity is a critical issue and attracts great interest due to consumer concern about food quality and safety. Authentic food is defined as the product that is precisely described by the label [1]. According to the Insurance Agency of Food Authenticity (IFAA), “authentication” is a process which unequivocally proves that the food is genuine [2]. In a global food market, consumers are interested in the origin and quality of the products they choose. Food authenticity concerns not only consumers, but the authorities, as well. Authentic foods have high economic significance both to the food industry and the national economy, especially in the case of high value export agricultural products, such as nuts.

Among nuts species, walnuts (*Juglans regia* L.) are a valuable nutritional source and play a dominant role in the Mediterranean diet. Walnut has been characterized as functional foods owing to its nutritional value and beneficial health effects [3,4]. The walnut kernel is a rich source of minerals, tocopherols, fatty acids, and phenolic compounds [5,6,7,8]. The cultivar and the geographical origin have been shown to affect the bioactive content of walnuts [3,9,10]. Walnut was cultivated in Europe as early as 1000 BC [4]. It has naturally diverged to several cultivars worldwide. Among the most commercial cultivars are: Chander, Hartley, Franquette, Mellanaise, Lara, Marbot, Mayette, Serr, Tulare, Sorento, etc. [11,12,13].

Even though walnuts are high-value agricultural products that provide energy, all the necessary nutrients ingredients and have beneficial health effects, they have not been adequately studied in terms of authenticity compared to other food products [14]. This gap has to be filled with the development of analytical methodologies that enable the determination of walnut constituents that could potentially be used as markers for the guarantee of walnuts genuineness. It is worth noting that the formation and development of a modern global food distribution system critically depends on the enforcement and implementation of quality controls. To this end, the European Union (EU) has established reference centers for authenticity and integrity of the agri-food chain to avoid fraudulent indices (EU, 2017/625, L95, 1-95). For agricultural products, such as walnuts, it is important to validate the country and area where the food was produced, as well as verify the cultivation practice.

The majority of the published articles focus mainly on the development of methodologies for the assessment of the mineral [5,15,16], lipid [6,17,18,19] or volatile profile [14,20] of walnuts [21,22]. Less is known about the minor phenolic fraction [8,23]. Phenolic constituents are secondary plant metabolites subjected to environmental modifications [8,24]. The analysis and evaluation of the phenolic profile of agricultural products provides valuable information about the quality characteristics among the plant species, and this is why the phenolic profile is investigated in authenticity studies [25]. Considering the chemical diversity of phenolic constituents (phenolic acids, flavonoids, lignans, stilbenes, and tannines), the generic analytical methodology for the determination of phenolic constituents involves extraction as the first step, and then analytical separation, identification and quantification follow. Liquid chromatography (LC) is the main technique used for the separation of phenolic compounds. Several methodologies using high pressure liquid chromatography (HPLC) coupled to UV or diode array detector (DAD), ultra-high pressure liquid chromatography (UHPLC) coupled to mass spectrometric detectors (MS), as well as high resolution mass spectrometric (HRMS) instruments which provide sufficient separation capacity and resolving power, are widely used in the analysis of phenolic analytes, as it has already been reviewed [8,26,27].

The further analysis of the chromatographic results with chemometric tools enhances the conclusions derived from the experimental data. The development of chemometric models enables the establishment of mathematical correlations in the data matrix, allowing the discovery of trends and behaviors among the samples. To such an end, the development of pattern recognition models allows the presentation of significant and non-evident information that is critical in authenticity issues [28]. Several authenticity studies have used unsupervised chemometric tools such as Principal Component Analysis (PCA) for exploratory data analysis, displaying the similarity of the observations as points in a map (score plot) [21,29,30]. Even though PCA finds correlations among the features successfully, it is affected by scale, and it may cause loss of information as compared to the original list of features. Supervised recognition techniques such as partial least squares–discriminant analysis (PLS-DA) overcomes the weaknesses of unsupervised methods and is considered unique in exploratory data analysis, providing good insight into the causes of discrimination via loadings and weights [31,32].

The objective of this work was to develop a rapid, accurate and selective HPLC-DAD analytical method for the determination of phenolic compounds in walnut samples belonging to the *Chandler* variety originating from Greece, Bulgaria and France and are available in the Greek market. A PLS-DA prediction model was developed to discriminate the samples and reveal characteristic markers responsible for their classification according to the geographical origin.

## 2. Materials and Methods

### 2.1. Chemicals and Reagents

Acetonitrile (ACN), HPLC grade, and methanol (MeOH) HPLC grade were purchased from Panreac–AppliChem (Darmstadt, Germany). Acetic acid 99% and trifluoroacetic acid (TFA) 99% were acquired by Sigma-Aldrich (Steinheim, Germany). Ultrapure water was provided by a Milli-Q ^®^ purification system (Millipore, Bedford, MA, USA). The following standard compounds: caffeic acid 98%, catechin 98%, diosmin 97%, epigallocatechin gallate 95%, ferulic acid 98%, gallic acid 98%, gallocatechin gallate 98%, kaempferol 97%, myricetin 98%, myricitrin 99%, p-coumaric 98% quercetin-3-o-glucoside 97%, rosmarinic acid 98%, rutin 98%, sinapic acid 95%, syringic acid 95%, vanillic acid 97%, and vanillin 99% were purchased by Sigma-Aldrich (Steinheim, Germany). Stock standard solutions were prepared for each analyte (1000 mg/L) in MeOH and stored at −20 °C in dark brown glass bottles.

### 2.2. Instrumentation

A quaternary low-pressure gradient HPLC–DAD system by Shimadzu (Kyoto, Japan) was used for chromatographic analysis. The HPLC system was equipped with: (a) an FCV-10ALVP mixing system, (b) a Rheodyne 7725i injection valve (Rheodyne, Cotati California, USA) geared with a 20 μL loop for sample injection, (c) an LC- 10ADVP pump equipped with a Shimadzu SCL-10ALVP System Controller, (d) an SPD-M10AVP photodiode array detector supplied with the soft- ware Lab Solutions-LC solutions by Shimadzu. For degassing the mobile phase, a DGU-10B de-gassing unit was used with helium. For the filtration of the mobile phases a glass vacuum filtration apparatus, acquired by Alltech Associates (Deereld, IL, USA), and nylon 0.2 μm membrane Filters (Alltech Associates, Chicago, IL, USA) were utilized. A vortexer purchased from FALC Instruments (Treviglio (BG), Italy) was used for sample agitation. For sample evaporation, a ReactiVap 9-port evaporator model 18,780 by Pierce (Rockford, IL, USA) was used. Centrifugation was carried out in a HermLe centrifuge, model Z-230 (B. HermLe, Gosheim, Germany). For sample filtration, Q-Max RR syringe filters (0.45 μm nylon membrane) were purchased from Frisenette ApS (Knebel, Denmark).

### 2.3. Chromatographic Analysis

Chromatographic separation was achieved on a C18 Fortis UniverSil column (250 mm × 4.6, 5 μm), supplied by Fortis Technologies Ltd. (Neston, UK), and operated at 30 °C. The binary mobile phase consisted of 1% acetic acid in water (A) and ACN (B) starting at a ratio of 95:5 (*v*/*v*), and then gradually increasing to 20% (Β) within 15 min, and then increasing to 50:50 (*v*/*v*) within the following 25 min, reaching a ratio of 10:90 (*v*/*v*) in the 45th min, and remaining stable for the following five min (t = 45–50 min). Then, the initial conditions (95% A, 5% B) were restored in the 55th min, and kept constant for five min to re-equilibrate the column for the next injection. Peak identification was carried out using the retention time (Rt) of the standard compounds, along with the spectral information provided by the DAD detector that operated over the range 250–400 nm. Peak monitoring and quantitation were performed at the maximum wavelength of each analyte. Peak identification was performed by using the data Rt_s_ and spectra from the DAD detector.

### 2.4. Walnut Samples

Twenty-six walnut samples of conventional farming commercially available to the Greek market, belonging to the Chandler cultivar were obtained. Ten Greek walnut samples were acquired from Thrace, Macedonia and Thessaly in Greece during November 2020. Ιmported walnuts from Bulgaria and France were supplied by traders who import walnuts from these countries and distribute them in the Greek market during the same time period (November 2020). Nine samples originating from Bulgaria, and seven samples originating from France were acquired. All samples were dried in a drying unit at 35 °C for 24 h and were then homogenized in a porcelain mortar prior to storage at −20°C, until analysis.

### 2.5. Extraction Optimization

A generic sample preparation protocol previously introduced by Pinasseau et al. [33] for the extraction of phenolic compounds was modified. Specifically, several extraction factors such as the extraction solvent, the ultrasound-assisted extraction (UAE) time and the extraction temperature were optimized following the well-established one-factor- at-a-time method (OFAT) [34]. In this respect, the recoveries (R%) of the previously reviewed as the most abundant phenolic compounds determined in walnuts [8]; namely gallic acid, vanillic acid, and syringic acid from the class of phenolic acids, as well as catechin, rutin, and quercetin-3-o-glucoside from the class of flavonoids, were calculated to evaluate the effect of extraction solvent (0.05% TFA in acetone, 0.05% TFA in methanol, 0.05% TFA in methanol: water at 60:40 ratio, *v*/*v*), the effect of extraction time (5–20 min), and the extraction temperature (4–40 °C). Briefly, 100 mg of homogenized walnuts was weighted in 2-mL eppendorf tubes and 0.5 mL of extraction solvent was added. The mixture was vortexed for 1 min, and then it was transferred in an ultrasonic bath to optimize extraction temperature and the extraction time. The samples were centrifuged for 10 min at 10,000 rpm. The supernatant was collected and dried under nitrogen flow and the extract was reconstituted in 0.2 mL of 1% acetic acid in water: acetonitrile (50:50, *v*/*v*).

### 2.6. Sample Preparation

In brief, 100 mg of homogenized walnuts was weighted in 2-mL eppendorf tubes and 0.5 mL of 0.05% TFA in methanol: water at 60:40 ratio (*v*/*v*), was added. The mixture was vortexed for 1 min, and then it was transferred in an ultrasonic bath at 25 °C for 10 min. Then, the samples were centrifuged for 10 min at 10,000 rpm. The supernatant was collected and dried under nitrogen flow and the extract was reconstituted in 0.1 mL of 1% acetic acid in water: acetonitrile (50:50, *v*/*v*). The diluent was filtered through 0.45 μm nylon syringe filters prior to injection in the chromatographic system.

### 2.7. Method Validation

Μethod validation was performed to estimate linearity selectivity, the limits of detection (LODs) and the limits of quantification (LOQs), within-day, and between-day accuracy and precision. Linearity studies were performed in triplicate using standard solutions and covered the working range of 2–20 μg/g. Seven-point calibration curves were constructed by plotting the peak areas versus concentration. For the calculation of the LODs and the LOQs the S/N ratio was monitored until a S/N ratio of 3:1 (LOD) and 10:1 (LOQ) was reached. For the evaluation of accuracy and precision a pool sample was prepared and spiked at low, medium, and maximum concentration levels of 0.5 μg/g, 10 μg/g, and 20 μg/g. Analysis was performed in triplicate. For the estimation of relative recoveries (R%) the found and added concentrations of the examined analytes were calculated (mean concentration found/concentration*100, R%), expressing accuracy. Precision was expressed as relative standard deviation (RSD%). Within-day precision (repeatability) was assessed in six replicates (*n* = 6), and between-days precision (reproducibility) was examined after performing triplicate analysis of spiked samples within three consecutive days (*n* = 3 × 3). To evaluate selectivity, five blank matrices were used and no interferences were observed in the same chromatographic window for both methodologies.

### 2.8. Chemometric Analysis

PLS-DA is a supervised pattern recognition technique used to find the appropriate class for each sample [32,35]. PLS-DA is a linear classification of the PLS regression that was initially used for regression task and evolved into a classification tool. A mathematical model is built and applied in the analysis of unknown samples to establish a correlation and classify them. The advantage compared to unsupervised classification techniques is that the samples belonging to each class are labelled, and in this respect the prediction model achieves the reduction of the dimensions knowing the lass labels.

A PLS-DA prediction model was developed using the MetaboAnalyst 5.0 platform [36], in an attempt to discover patterns in the quantitative data of the determined phenolic compounds and predict the geographical origin of the analyzed samples, establishing the most important compounds used for the classification as characteristic markers.

## 3. Results

### 3.1. Extraction Optimization Results

The effects of the extraction solvent, the UAE time, and extraction temperature were studied following the OFAT method [34]. The extraction recoveries (R%) of gallic acid, vanillic acid, and syringic acid from the class of phenolic acids, and catechin, rutin, and quercetin-3-o-glucoside from the class of flavonoids, were calculated to evaluate the effect of each parameter on the extraction efficiency.

**Figure 1:**
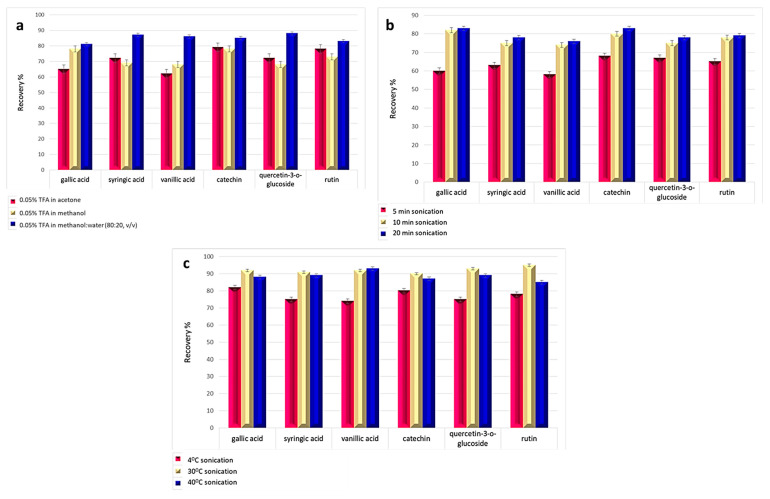
Effect of (**a**) extraction solvent; (**b**) extraction time, (**c**) extraction temperature on the extraction efficiency.

The extraction solvent was the first factor to be studied. According to the results presented in Figure 1a, the higher recoveries were obtained for 0.05% TFA in methanol:water at 60:40 ratio, *v*/*v*). The findings are in accordance with the literature, since the majority of the studies report the use of acidified mixtures of methanol:water [8]. The second factor that was evaluated was the time of the extraction in an ultrasonic bath. According to the results presented in Figure 1b, the increase in the extraction time increased the recoveries of all the analytes. The difference in the extraction efficiency between 10 min of sonication and 20 min was less than 4% and, in an attempt to minimize extraction time, the extraction time of 10 min was selected as the optimum for the next experiment. The last parameter that was evaluated was the extraction temperature. Three different temperatures were tested (4 °C, 30 °C and 40 °C). The highest recoveries (≥90%) were obtained at 30 °C, and this temperature was selected as the optimum. The extraction at low temperature (4 °C) was not sufficient enough resulting in low recoveries over the range 72–81%. On the other hand, the further increase of temperature at 40 °C resulted in a slight decrease of the obtained recoveries, compared to the results obtained at 30 °C, which could be explained due to the degradation of phenolic constituents at higher temperatures [37].

### 3.2. Method Validation Results

All the analytical parameters of the developed HPLC-DAD methodology for the determination of phenolic compounds in walnuts (i.e., the calibration curves and linear range, the determined coefficients (r^2^), the calculated LODs, and LOQs, accuracy and precision are summarized in Table 1. According to the results, r^2^ ranged from 0.991 to 0.999 establishing the good linearity of the method. The LOQs were found to range between 0.30 μg/g (gallic acid) and 1.44 μg/g (myricetin), while the LODs were calculated equal to 0.10 (gallic acid)–0.48 μg/g (myricetin). The RSD% of the within-day (*n* = 6) and between-day assays (*n* = 3 × 3) was lower than 6.3, and 11.1, respectively, showing adequate precision. The accuracy was assessed by means of relative percentage of recovery (%R) at low, medium, and maximun concentration levels (2, 10, 20 μg/g), and the results were acceptable, ranging from 86.4 (sinapic acid, at 20 μg/g concentration level) to 98.4% (caffeic acid, at 20 μg/g concentration level) for within-day assay (*n* = 6) (Table 2), and from 90.1 (gallocatechin gallate, at 20 μg/g concentration level) to 100.6% (gallic acid) for between-day assay (*n* = 3 × 3) (Table 3).

### 3.3. Walnut Analysis

**Figure 2:**
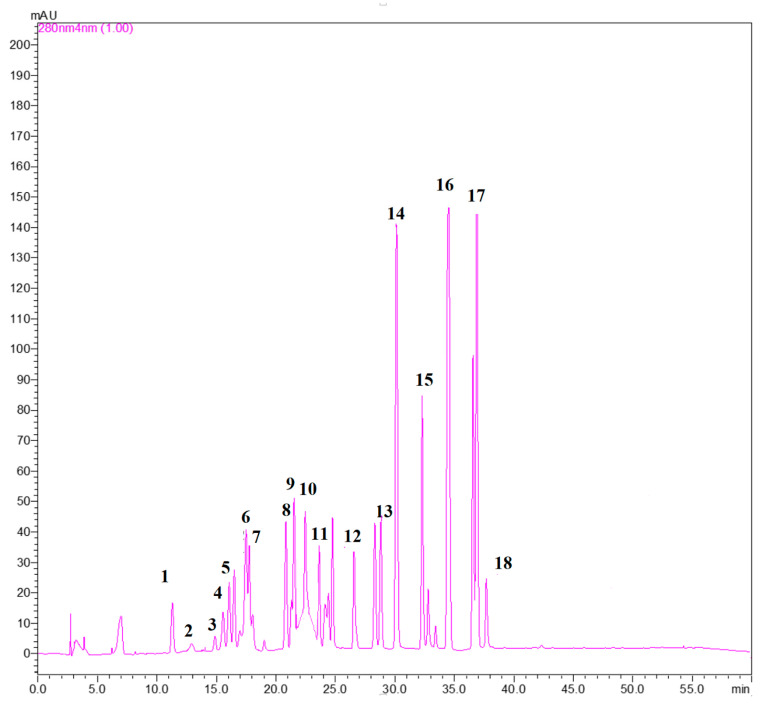
Characteristic chromatogram of a walnut sample spiked with a standard mixture at 2 μg/g; monitored at 280 nm.

The optimized and validated HPLC-DAD analytical method was applied in the analysis of real samples. Twenty-six walnut samples belonging to the *Chandler* variety originating from Bulgaria, Greece, and France were analyzed and eighteen phenolic compounds were determined, in total. Those were: caffeic acid, ferulic acid, gallic acid, p-coumaric acid, rosmarinic acid, sinapic acid, syringic acid, and vanillic acid, from the class of phenolic acids, and catechin, diosmin, epigallocatechin gallate, gallocatechin gallate, kaempferol, myricetin, myricitrin, quercetin-3-o-glucoside, rutin, and vanillin from the class of flavonoids. Table 4 lists the identified phenolic compounds along with their retention times Rts and maximum absorption wavelengths (*λ*, nm). Figure 2 presents a characteristic chromatogram of a walnut sample spiked with a standard mixture at 2 μg/g and monitored at 280 nm.

#### Quantification Results

All samples were analyzed in triplicate (*n* = 3). The identified analytes were quantified using their maximum absorption wavelengths. The quantification ranges of each phenolic compounds as well as the mean values (±SD) are presented in Table 5. The quantification results are in accordance with those previously reported by Slatnar et al. [38], Ho et al. [39], and Vu et al. [40].

Eighteen phenolic compounds were determined proving that walnut kernels are rich in phenolics. Variations in the phenolic concentration ranges have already been reported in walnuts of different varieties [38,39,40], but there are limited reports concerning the effects of the geographical origin on the concentrations of the phenolic compounds.

The highest mean concentration in Greek walnuts was observed for myricetin (125.3 μg/g), and the second highest mean value was observed for epigallocatechin gallate (121.6 μg/g), while p-coumaric acid was ranked third with a mean concentration equal to 89.5 μg/g, and catechin followed with a mean concentration of 81.5 μg/g. As for Bulgarian walnuts, the highest mean concentration was observed for epigallocatechin gallate (114 μg/g). The second most abundant phenolic compound was myricetin with a mean value equal to 85.1 μg/g, and catechin followed with a mean concentration of 75.1 μg/g. As far as French walnuts are concerned, the highest mean concentration was observed for myricetin (131.6 μg/g). The second highest mean concentration was reported for p-coumaric acid (85.3 μg/g), and syringic acid followed with a mean concentration equal to 57.7 μg/g. High concentrations were observed for gallic acid, ferulic acid vanillic acid, from the class of accordingly to Vu et al. [40]. Sinapic acid presented high concentrations, as well, and the highest average concentration was determined in walnuts originating from Bulgaria (72.6 μg/g). Relatively lower concentrations were determined for caffeic acid, compared to the rest of the phenolic acids, which presented mean values of 2.05 μg/g, 4.25 μg/g, and 3.65 μg/g, for Greek, Bulgarian, and French walnuts, respectively. As for the rest of the flavonoids, the highest average concentration of diosmin (5.16 μg/g) was observed in Bulgarian walnuts, and the lowest in French (3.06 μg/g). The highest mean concentrations for vanillin were calculated in Greek walnuts (5.55 μg/g). Bulgarian walnuts were rich in kaempferol (6.32 μg/g), while French walnuts showerd higher values for gallocatechin gallate (7.02 μg/g). The obtained concentrations of querce-tin-3-o-glucoside were similar to other varieties of walnuts, such as Black and English [40].

### 3.4. PLS-DA Model

**Figure 3:**
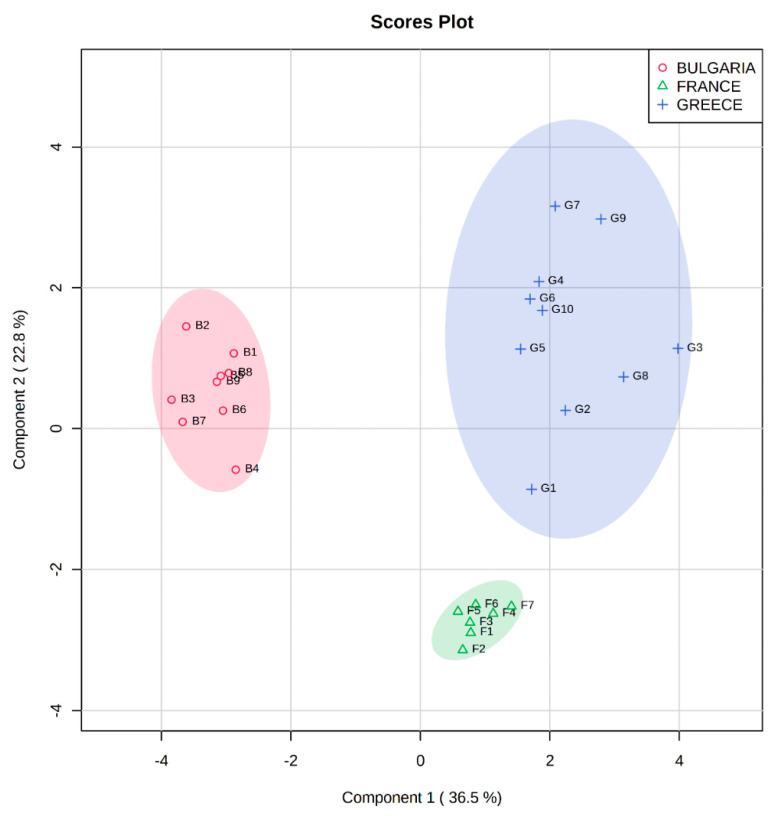
PLS-DA prediction model score plot showing the discrimination of the walnuts originating from Bulgaria (B1−B9) in red color, France (F1−F7) in green color, and Greece (G1−G10) in blue color.

**Figure 4:**
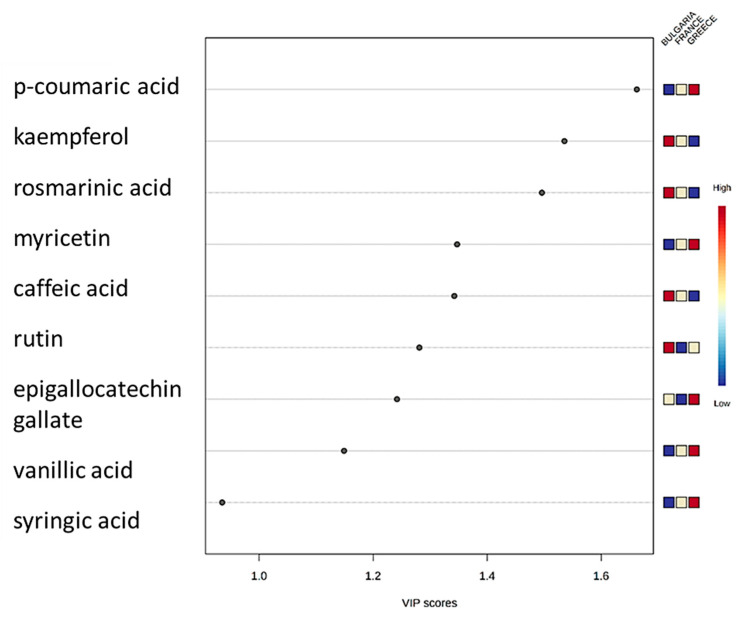
VIP scores of the most important features causing greater variation in the developed PLS-DA model.

A PLS-DA model was developed using the MetaboAnalyst platform [36]. The chemometric model classified the samples according to the geographical origin (Bulgaria, Greece, and France) successfully with an explained variance of 59.5% within the first two dimensions. The score plot of the developed PLS-DA model is presented in Figure 3, showing the clustering of three individual groups of walnut samples. The colored areas around the samples (red for Bulgaria, blue for Greece, green for France) represent the 95% confidence region of the replicates. Variable importance in projection (VIP) algorithm was used to estimate the significance of each variable in projection used to build the PLS-DA model. The VIP scores of the variables show their contribution in the final model. According to Mehmood et al. [41], the cut-off value of above 0.83 was used for the VIP score. The Figure 4 shows the most important features with calculated VIP scores above 0.83. p-Coumaric acid, kaempferol, rosmarinic acid, myricetin, caffeic acid, rutin, epigallocatechin gallate, vanillic acid, and syringic acid were selected as the most important markers responsible for the discrimination between the walnut samples originating from different countries. According to Figure 4, the compounds that cause greater variation and are characteristic for each geographical origin are marked in red. In this respect, p-coumaric acid, myricetin, epigallocatechin gallate, syringic acid and vanillic acid cause greater variation in the Greek samples; while kaempferol, rosmarinic acid, caffeic acid and rutin are characteristic markers of the Bulgarian walnuts. The compounds marked in yellow cause slightly lower variation to each category of samples, and those marked in blue cause the lowest variation.

**Figure 5:**
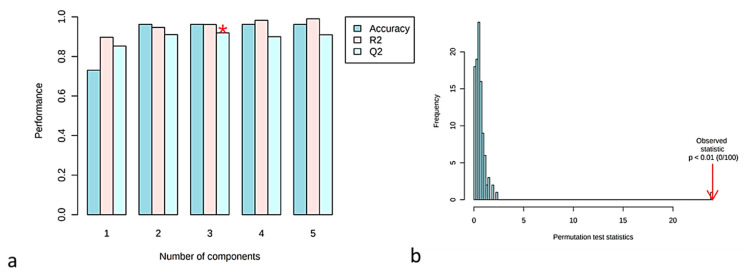
(**a**) Cross validation parameters of the developed PLS-PDA model with the prediction error measure: accuracy, R2, Q2 (The accuracy = 0.96, was obtained from the third component shown with asterisk.); (**b**) Permutation test statistics (100 random permutations).

For validation, the Leave-One-Out Cross-Validation (LOOCV) method was applied using five components. The goodness of fit (R^2^ = 0.99) and the predictability of the model (Q2 = 0.90) values suggest that this is a PLS-DA model with strong predictive power (Figure 5a). The accuracy = 0.96, was obtained from the third component, shown in Figure 5a with asterisk. Permutation test statistics (100 random permutations) were calculated and the results verified that the walnut samples significantly differed (with one sample *t*-test with *p*-value < 0.01) from each other [42] (Figure 5b).

## 4. Conclusions

A novel HPLC-DAD method was developed and optimized for the determination of phenolic compounds in 26 walnut samples of the Chandler variety originating from Bulgaria, Greece, and France. Overall, eighteen phenolic compounds were determined (caffeic acid, catechin, diosmin, epigallocatechin gallate, ferulic acid, gallic acid, gallocatechin gallate, kaempferol, myricetin, myricitrin, p-coumaric acid, quercetin-3-o-glucoside, rosmarinic acid, rutin, sinapic acid, syringic acid, vanillic acid, and vanillin) in walnut samples. The quantification results were further analyzed with chemometrics, and a PLS-DA model was developed and successfully classified the walnut samples based on their geographical origin, with the first two dimensions explaining the 59.5% of the total variance. p-coumaric acid, kaempferol, rosmarinic acid, myricetin, caffeic acid, rutin, epigallo-catechin gallate, vanillic acid, and syringic acid were proposed as markers responsible for the discrimination between the walnut samples of different geographical origins.

This work has made progress towards the phenolic characterization of walnuts of the *Chandler* variety originating from Bulgaria, Greece, and France highlighting that the geographical affects the phenolic profile and proposing a robust PLS-DA model that could be used for the prediction of the geographical origin in authenticity studies and the detection of fraudulent indices.

## Figures and Tables

**Table 1:** HPLC-DAD method analytical parameters.

Compound	Calibration Equation (Linear Range: 2–20 μg/g)	r^2^	LOD (μg/g)	LOQ (μg/g)
caffeic acid	y = 110028.53x + 5532	0.992	0.15	0.45
catechin	y = 12565x + 1204	0.994	0.30	0.90
diosmin	y = 14007.23x + 1353	0.991	0.20	0.60
epigallocatechin gallate	y = 19304x − 1212	0.996	0.40	1.20
ferulic acid	y = 88455x + 4328	0.994	0.25	0.75
gallic acid	y = 10562x − 11.8	0.997	0.10	0.30
gallocatechin gallate	y = 2004x − 1450	0.996	0.45	1.35
kaempferol	y = 1878x + 2242	0.998	0.45	1.35
myricetin	y = 19502x + 1026	0.997	0.48	1.44
myricitrin	y = 20521x + 1408	0.996	0.32	0.96
p-coumaric acid	y = 107385x + 4325	0.999	0.25	0.75
quercetin-3-o-glucoside	y = 17542x + 2404	0.997	0.28	0.84
rosmarinic acid	y = 1508x + 48.3	0.998	0.15	0.45
sinapic acid	y = 107562x + 2385	0.994	0.24	0.72
rutin	y = 20008x + 423	0.993	0.34	0.96
syringic acid	y = 105424x + 4728	0.995	0.25	0.75
vanillic acid	y = 65405x + 1125	0.998	0.20	0.60
vanillin	y = 138452x − 4585	0.994	0.35	1.05
caffeic acid	y = 125475x + 1842	0.993	0.20	0.60

LOD: limit of detection, LOQ: limit of quantitation.

**Table 2:** %Recoveries (%R, *n* = 6) for the evaluation of repeatability.

Compound	%R Low Conc. Level (2 μg/g)	%RSD	%R Medium Conc. Level (10 μg/g)	%RSD	%R Maximum Conc. Level (20 μg/g)	%RSD
caffeic acid	91.4	4.7	92.5	2.2	98.4	2.5
catechin	90.5	5.5	94.4	3.1	91.2	1.7
diosmin	96.4	3.1	95.6	5.1	93.6	3.9
epigallocatechin gallate	92.3	1.3	90.8	4.4	94.1	4.2
ferulic acid	91.7	2.5	91.7	6.1	90.8	5.1
gallic acid	92.1	3.6	92.4	3.1	92.1	6.3
gallocatechin gallate	93.3	4.1	91.6	2.4	94.6	5.5
kaempferol	91.8	5.2	89.7	5.3	92.8	3.7
myricetin	90.9	4.3	92.4	1.8	93.5	4.9
myricitrin	94.5	1.9	93.2	2.6	94.3	3.4
p-coumaric acid	95.6	2.5	91.4	3.1	91.1	4.8
quercetin-3-o-glucoside	93.0	2.7	95.2	5.4	93.1	2.7
rosmarinic acid	92.2	3.5	93.7	4.9	89.4	5.6
sinapic acid	91.1	2.8	92.4	6.3	86.4	2.8
rutin	94.2	3.6	93.5	3.9	92.6	5.7
syringic acid	93.1	5.4	92.6	2.7	92.3	6.2
vanillic acid	92.8	6.2	90.7	1.6	94.5	4.3
vanillin	93.7	5.1	92.4	3.5	93.9	2.5

Conc.: Concentration.

**Table 3:** %Recoveries (%R *n* = 3 × 3) for the evaluation of intermediate precision.

Compound	%R Low Conc. Level (2 μg/g)	%RSD	%R Medium Conc. Level (10 μg/g)	%RSD	%R Maximum Conc. Level (10 μg/g)	%RSD
caffeic acid	95.3	5.3	97.2	7.2	93.2	7.2
catechin	92.8	7.1	96.5	5.4	92.4	6.8
diosmin	97.4	8.8	93.1	4.8	91.5	5.5
epigallocatechin gallate	93.5	7.9	91.4	9.1	94.3	5.9
ferulic acid	97.1	6.3	95.8	7.4	92.2	6.4
gallic acid	98.2	6.8	100.6	5.2	95.6	7.1
gallocatechin gallate	91.9	7.2	97.8	6.6	90.1	6.9
kaempferol	95.1	9.4	93.5	5.4	92.4	7.3
myricetin	93.7	8.3	94.2	8.8	93.3	8.1
myricitrin	92.2	7.6	96.6	7.1	96.1	6.5
p-coumaric acid	91.6	6.7	95.1	11.1	95.5	7.1
quercetin-3-o-glucoside	94.4	8.5	98.3	5.9	94.7	6.8
rosmarinic acid	92.3	9.1	95.7	8.7	92.1	5.2
sinapic acid	91.7	7.5	97.5	9.1	91.5	3.4
rutin	92.2	7.1	98.2	6.7	95.5	9.2
syringic acid	90.5	8.3	91.1	5.8	90.7	6.5
vanillic acid	93.1	8.4	95.3	7.3	91.1	5.8
vanillin	94.4	5.7	92.4	6.4	92.1	7.5

Conc.: Concentration.

**Table 4:** Retention time and maximum absorption wavelength of the phenolic analytes determined in walnuts.

Compound	Rt	*λ* (nm)
gallic acid	11.1	278
gallocatechin gallate	12.6	285
catechin	14.6	280
vanillic acid	15.5	260
epigallocatechin gallate	17.4	280
syringic acid	18.1	274
rutin	19.3	353
myricitrin	20.6	360
p-coumaric acid	21.4	270
vanillin	22.6	278
sinapic acid	23.5	260
quercetin-3-o-glucoside	26.5	365
diosmin	28.8	345
ferulic acid	29.8	293
caffeic acid	32.2	284
myricetin	34.3	370
rosmarinic acid	37.1	272
kaempferol	37.9	360

Rt: retention time.

**Table 5:** Quantification results of the phenolic compounds determined in walnuts originating from Greece, Bulgaria, and France (samples analyzed in triplicate, *n* = 3).

Origin	Greece		Bulgaria		France	
Compound	Concentration Range (μg/g)	Mean Value (μg/g ± SD)	Concentration Range (μg/g)	Mean Value (μg/g ± SD)	Concentration Range (μg/g)	Mean Value (μg/g ± SD)
caffeic acid	LOQ–2.56	2.05 ± 0.28	2.67–5.58	4.25 ± 0.54	2.35–4.42	3.65 ± 0.33
catechin	78–148	81.5 ± 5.24	34–122	75.1 ± 2.35	4.21–75.3	34.2 ± 6.05
diosmin	LOQ–23.8	4.32 ± 0.14	LOQ–22.1	5.16 ± 0.39	2.25–8.32	3.06 ± 0.21
epigallocatechin gallate	75.1–173.4	121.6 ± 14.9	18.7–125.6	114 ± 11.5	40.8–63.9	51.6 ± 7.66
ferulic acid	25.1–168.4	67.4 ± 7.52	31.2–86.4	55.9 ± 3.35	22.1–29.8	23.5 ± 2.11
gallic acid	4.21–75.6	45.3 ± 6.18	20.3–78.5	57.5 ± 8.42	2.14–45.8	25.6 ± 5.24
gallocatechin gallate	3.20–4.64	4.12 ± 0.08	2.24–5.36	4.78 ± 0.23	5.78–8.20	7.02 ± 0.69
kaempferol	LOQ-5.21	2.04 ± 0.32	5.12–9.20	6.32 ± 2.54	2.75 ± 4.25	3.04 ± 0.41
myricetin	73.4–148.23	125.3 ± 16.6	26.1–98.8	85.1 ± 9.27	105.4–178.3	131.6 ± 27.8
myricitrin	2.30–3.65	3.08 ± 0.78	2.18–2.99	2.36 ± 0.13	2.65–3.56	2.85 ±0.22
p-coumaric acid	83.1–107.4	89.5 ± 12.2	29.4–40.5	31.3 ± 2.35	72.1–93.5	85.3 ± 7.72
quercetin-3-o-glucoside	3.31–6.12	4.78 ± 0.54	5.61–0.24	6.28 ± 0.75	2.98–3.65	3.14 ± 0.09
rosmarinic acid	2.85–19.6	7.85 ± 1.05	35.7–56.4	42.6 ± 2.37	18.3–30.4	23.6 ± 2.33
rutin	21.5–35.4	27.4 ± 7.22	53.2–66.8	57.7 ± 3.85	12.5–28.8	21.4 ± 3.44
sinapic acid	LOQ–83.4	35.4 ± 3.31	53.5–121.8	72.6 ± 11.3	20.7–47.8	31.1 ± 4.06
syringic acid	32.1–75.4	46.2 ± 3.66	19.3–58.6	37.2 ± 1.89	53.1–67.8	57.7 ± 5.32
vanillic acid	7.53–95.6	62.7 ± 5.65	LOQ–73.5	65.4 ± 7.04	7.58–41.2	37.3 ± 5.32
vanillin	1.81–8.56	5.55 ± 0.89	1.71–4.42	3.78 ± 0.65	1.85–5.56	4.68 ± 1.85
